# The Endoscopic Treatment of Chronic Urethritis

**Published:** 1899-06

**Authors:** W. L. Champion

**Affiliations:** Atlanta, Ga.; Ex-President Atlanta Society of Medicine; Member of the American Medical Association, Etc.


### THE ENDOSCOPIC TREATMENT OF CHRONIC URETHRITIS.*

BY W. L. CHAMPION, M.D., ATLANTA, GA.

Ex-President Atlanta Society of Medicine; Member of the American Medical Association, Etc.

It is my desire in presenting this short paper to state a few facts in regard to the treatment of chronic urethritis which I hope will result in the more general use of the endoscope.

A careful study of the lesions of chronic urethritis through the endoscope will convince the most skeptical of the value of direct local treatment in these conditions. From practical experience I can safely say that a large percentage of inflamed urethras remain uncured for months, and frequently years, when the proper endoscopic treatment would perfect a speedy cure. After several years' use of the endoscope in treating these conditions, it has been my good fortune to relieve many suffering individuals, if not from pain, from the mental anxiety produced on account of a chronic urethral discharge, or the probability of a stricture in the near future.

As stated by Klotz, "Many patients can be found who have had their chronic gonorrhea treated by internal medicines, by the usual injections administered by themselves, by deep injections made by the surgeon, by irrigation, by sounds, and even by an abundant urethrotomy, and who still have a mucous or purulent discharge. In such cases the presence of numerous filaments in

*Read at the meeting of the Georgia Medical Association, Macon, April, 1899.

the urine makes the existence of some pathological condition almost a certainty, and the desire to look into the urethra and find the cause of the continuous discharge is but natural, and is justified by the fact that in the greater number of cases pathological conditions are easily found."

It is not the object of this paper to go into the minutiæ of the pathology of chronic urethritis, but only in a general way state those conditions we can see and which will respond to treatment through the endoscope. It is only after careful study and some experience with the use of the endoscope that the various pathological conditions can be recognized. We will have to bear in mind that the healthy urethra in the prostatic portion has a moist and smooth surface of a decided red color which gradually becomes paler along the urethral canal until we reach the fossa navicularis, when the appearance becomes almost white. On account of the urethra being a closed canal composed partly of muscular tissue, its normal appearance through the endoscope is funneled-shaped.

Now, after a thorough knowledge of the appearance of the healthy urethra, any variation from normal can be readily recognized—such as intense congestions, granulations, ulcers, epithelial thickenings, warty growths, and involvement of the follicles and glands. As stated, in the normal condition the urethra has a funnel shape. The shape or size of the funnel is altered in proportion to the amount of inflammation in the mucous membrane and submucous tissue. When highly inflamed the funnel is obliterated and the membrane bulges into the tube, which is also the case with papillomatous growths, bnt these can be readily recognized and removed through the tube. In stricture of the urethra the endoscope is of little value, except in cases of tight stricture it may be of service in finding the opening to introduce a filiform bougie.

There is frequently a subepithelial thickening and granular condition of the mucous membrane which produces a slight narrowing of the canal that is diagnosed and occasionally operated upon as cicatricial stricture of large calibre. Strictures of this kind, if they can be called strictures, are the ones that give such quick and happy results from direct applications through the endoscope. The granular condition that remains after a stricture has been dilated or cut to its full size will respond more promptly to local treatment with the endoscope than other means. While the sound and deep injection syringe were originally, and are yet used for the relief of these conditions, it is impossible to know the condition the urethra is left in when the patient is dismissed.

In chronic urethritis not due to cicatricial stricture my experience has been that in the majority of cases the seat of inflammation is in the middle urethra, or more properly speaking, the lower portion of the spongy. That the spongy portion of the canal is more frequently involved than the membranous or prostatic there can be no doubt, but I am convinced that in every case of chronic urethritis, and what I mean by chronic is an inflammation that has extended over a period of eight or nine weeks, the posterior urethra is more or less involved. This can be demonstrated by taking a patient with a few ounces of urine in his bladder, whose urethra is inflamed and bladder is not involved, and irrigate the anterior urethra thoroughly, and then have the patient pass his urine in a glass, and invariably shreds or filaments will be noticed.

The endoscope should not be used when the urethra is acutely inflamed, or so long as the urine appears cloudy from an abundance of mucus. It is stated a sound should be passed upon a patient who has never had an instrument used in the urethra before using the endoscope. This is advised, I suppose, to accustom the urethra to the use of instruments. I cannot see why an endoscopic tube properly used should be any more liable to produce untoward results than a sound. I never hesitate to use an endoscope the first time I see a patient if it is indicated, and have never yet had any bad results to follow. Of course, if the urethra is strictured, sounds should be used to dilate or urethrotomy, as the case may indicate. It is proper to know the canal is free of stricture before commencing treatment with the endoscope. If the meatus will not admit a tube of sufficient calibre for examination and treatment it should be cut to the full size of the urethra.

The endoscope which I use, and I believe one of the best, is W. K. Otis's, with Klotz's endoscopic tubes. It is a very simple matter, and practically without pain, to introduce an endoscopic tube the entire length of the spongy urethra, but usually very difficult, and frequently impossible, to insert the straight tube into the membranous and prostatic portions of the canal so as to make a satisfactory examination or proper local treatment. From reading the articles written upon the use of the endoscope we would judge that it was a very easy matter to insert the endoscopic tube into the membranous and prostatic urethra. Now, any of you who have ever examined the posterior urethra through the endoscope or tried to pass a straight sound into the bladder know how difficult it is, and that it is very painful to the patient.

As stated above, in the majority of cases of chronic urethritis the diseased area is in the spongy portion of the canal, but we frequently find the posterior urethra involved to such a degree that it is necessary to use local treatment. I insert the straight tube into the posterior urethra for examination and treatment, but usually with difficulty and in a limited number of cases, on account of extreme sensibility and nervous condition of the patient.

My associate, Dr. J. A. Childs, and myself, using the straight endoscopic tube in every-day practice, the only kind in use, so far as I know, and seeing how difficult and unsatisfactory it is in treating the membranous and prostatic portions of the urethra, decided to have a tube made that could be introduced very easily and without pain into the posterior urethra. The picture I show here is a fair representation of the instrument as devised by Dr. Childs. The shape of it is very much the same as the Leiter cystoscope, which can very readily be inserted into the bladder. The tube is introduced when curved, or in position marked A. The screw at the top is turned and the tube becomes perfectly straight, as represented by B. The obturator is then removed and Otis's electric endoscope is attached, when we can get a good view of the entire urethra or the walls of the bladder. This instrument is not intended to take the place of the straight tube in making examinations or treating the anterior urethra, as the straight tube can be handled more easily and is shorter, while it should always be remembered that the shorter the tube the better view we get.

Before making an examination or treating the urethra, the bladder should be emptied. Any surgeon's chair or an ordinary table will answer every purpose to place the patient upon. A head mirror to reflect the light will not give satisfaction. The light that I use is derived from the current that is used by the city, it first having passed through Wotton's Surgical Tranfsormer, so as to regulate the amount of current needed. A tube large enough to get a good view of the canal should be used, but not to the full size of the urethra, as it will by compression produce an anemic condition which will alter the appearance of the membrane. From a 26 to a 30 French tube will answer every purpose for ordinary examination or treatment. Cocain should not be used in the urethra before using the endoscope; it alters the appearance of the membrane, and, in my opinion, it is unnecessary, certainly from the standpoint of relieving pain. Glycerine should be used as a lubricant for the tube, as vaseline will interfere with the action of the drugs used in treatment.

Small wire applicators to hold the pledget of cotton for removing secretions and applying different medical agents are necessary. If the urethra is in a very highly granular condition, after inserting the tube there may be a slight hemorrhage that will temporarily interfere with the careful examination or proper treatment of the diseased surface, but at the next treatment there is usually such improvement that there will not be a recurrence of the hemorrhage.

In regard to drugs used, there are quite a number that are very useful, such as Lugol's solution, carbolic acid and iodine, nitrate of silver, tannin, bichloride of mercury, sulph. copper, ichthyol, solutions of iron, and many others. While we may select from the above list the drug best suited for each individual case, I use nitrate of silver more than any other, and with better results. It is best when using the silver solution to commence with about 20 grains to the ounce and increase to 60 grains to the ounce. The surface touched with the solution turns white, and there is usually a slight pain that lasts for several minutes. It is well to place a pledget of cotton over the meatus after the application, and tell the patient there will be an increase of discharge, which will usually pass off within 24 hours. It is best not to repeat the application oftener than every six or seven days. The day after the application the urethra should be irrigated with a solution of permanganate of potash 1 to 6,000. This relieves the irritation and slight discharge that is usually seen. There is very little danger of producing epididymitis, urethral fever, or any other complication from the use of the endoscope. I have never seen the slightest bad result follow its use.

I could report quite a number of cases of chronic urethritis that had extended over a period of from one to fifteen years that have been permanently cured by the use of the endoscope, but I will not bore you with the history. It might be well to state that many of these cases had been intelligently treated ; the stricture, if one had been present, was properly dilated or cut; the meatus was incised, the urethra injected or irrigated, but the chronic inflammatory condition remained, which was promptly relieved by proper endoscopic treatment.

It would be folly to hold that the endoscope is necessary to the successful treatment of every case of chronic urethritis, but the number of cases, which are not a few, that respond to this treatment after other means have failed, certainly demonstrates the value of the endoscope.

References: Prince A. Morrow, M.D., "A System of Genito-urinary Diseases, Syphilology and Dermatology," White and Martin, "Genito-urinary and Venereal Diseases."

*701, 702 and 703 Prudential Building.*

